# BiMSGP: A Bidirectional Mamba-Based Model for Scalable and Accurate Genomic Prediction in Plants

**DOI:** 10.3390/genes17091156

**Published:** 2026-09-20

**Authors:** Qingjie Liu, Xinwei Yao, Jinyan Ma, Huilin Cheng, Huahao Zhou, Licong Ding, Songyang Huai, Xu Liu

**Affiliations:** 1 College of Computer Science and Technology, Nanjing University of Aeronautics and Astronautics, Nanjing 211106, China; 2 China Nanhu Academy of Electronics and Information Technology, Jiaxing 314001, China; 3 Academy for Advanced Interdisciplinary Science and Technology, Zhejiang University of Technology, Hangzhou 310014, China; 4 Applied Mathematics, College of Informatics, Huazhong Agricultural University, Wuhan 430070, China

**Keywords:** BiMSGP, bidirectional Mamba, selective state space model, genomic prediction, complex traits, plant breeding, genomic selection

## Abstract

**Background/Objectives:** Accurate prediction of complex traits from high-dimensional genomic markers remains difficult in plant breeding. Linear models have limited capacity for non-linear interactions, and many deep learning models scale poorly on long marker sequences. This study introduces BiMSGP, a bidirectional Mamba-based model for scalable genomic prediction. **Methods:** BiMSGP extends a selective state space model with forward and backward processing so that each marker can integrate sequence context from both directions while retaining linear computational complexity; this is a modeling device for long-range marker dependencies, not a demonstration of biological epistasis. Regression was evaluated on Wheat599, Wheat2000, and SoyBase, and classification on SoyBase, using 10-fold cross-validation with shared fold assignments and no inner validation split; the held-out fold was used for checkpoint selection, learning-rate adjustment, and reporting. Ablation studies compared bidirectional and unidirectional stacks and different depths. Exploratory paired tests of fold-wise PCC differences were used to distinguish numerical rank from a paired difference under this protocol. **Results:** Under the reported evaluation protocol, BiMSGP attained the numerically highest mean Pearson correlation coefficient (PCC) in all four Wheat599 environments, six of eight Wheat2000 traits, and five of seven quantitative SoyBase traits. Several of these numerical ranks did not have exploratory $p_{t}<0.05$ against the strongest baseline; GBLUP had a higher mean PCC for SoyBase oil ($p_{t}=0.025$). Shallow bidirectional designs had higher mean PCC than deeper and unidirectional variants on Wheat599 Environment 1. Training cost was higher than that of lightweight baselines on larger datasets, but inference remained in the millisecond range. **Conclusions:** Under this protocol, bidirectional selective state space modeling yielded competitive mean scores with a higher training cost than lightweight baselines. The scores are exploratory and may be optimistic; they are not unbiased estimates of generalization. Evaluation that separates model selection from assessment, and tests in independent populations, years, or environments, remains future work.

## 1. Introduction

Accurately predicting complex phenotypic traits from genomic data is a central challenge in plant breeding and genetics, with significant implications for accelerating genetic gain and improving crop productivity [1,2,3,4,5,6]. Genomic prediction (GP) uses genome-wide markers to estimate breeding values or phenotypic outcomes before all candidates are phenotyped. Its practical application is complicated by the high-dimensional, low-sample-size structure of many breeding datasets, in which the number of markers greatly exceeds the number of individuals [7,8,9,10].

Traditional statistical approaches, such as genomic best linear unbiased prediction (GBLUP) and ridge-regression BLUP, are computationally efficient and remain strong baselines [11,12,13]. Their predominantly additive formulations, however, can have limited capacity to represent non-linear effects and interactions that contribute to complex traits [14,15]. Comparative studies have consequently explored machine- and deep-learning alternatives, while also showing that performance depends strongly on trait architecture, sample size, and model tuning [9,16,17]. Convolutional models such as DeepGS and DLGWAS learn local marker patterns, and newer systems including DNNGP, SoyDNGP, and DeepCCR extend deep genomic prediction to multi-omics, soybean, and large rice populations [6,18,19,20,21]. Fixed local receptive fields and the cost of modeling long marker sequences nevertheless remain important constraints.

Long-range sequence models have demonstrated the value of integrating distant genomic context, from hybrid convolutional–recurrent networks and long-context regulatory models to genomic language models [22,23,24]. State space models provide another route to long-context modeling: HiPPO and S4 established efficient structured-memory formulations, and Mamba introduced input-dependent selective state spaces with linear sequence complexity [25,26,27,28]. Building on this foundation, we propose **BiMSGP** (**Bi**directional **M**amba-based model for **S**calable and Accurate **G**enomic **P**rediction), a bidirectional Mamba architecture specifically designed for plant genomic prediction.

By processing genomic marker sequences in both forward and backward directions, BiMSGP enables each marker to incorporate contextual information from both sides of the sequence. This bidirectional scan can help the model use long-range marker dependencies; it is not direct evidence of biological epistasis between distant loci. The model retains the linear-complexity scan of Mamba.

The main contributions of this work are as follows:

1. We propose BiMSGP, a bidirectional Mamba-based architecture tailored for genomic prediction. Ablation studies on Wheat599 show that shallow bidirectional stacks had a higher mean PCC than deeper and unidirectional variants. A residual-off variant was an ablation finding on that panel and was not used in the main comparison tables.
2. We evaluate BiMSGP on three datasets of increasing scale using within-dataset 10-fold cross-validation: Wheat599 (599 accessions and 1279 markers), Wheat2000 (2000 accessions and 33,709 markers), and SoyBase (trait-specific *n* up to 15,878 accessions and 42,195 SNPs in the processed matrix). The held-out fold was also used for checkpoint selection, so the reported scores are exploratory under this protocol. We report numerical ranks together with exploratory paired tests of fold-wise PCC differences and do not interpret those scores as unbiased estimates of generalization.
3. We analyze the accuracy–efficiency trade-off of BiMSGP. The model has far more parameters than lightweight baselines (42 M versus 81 K for DeepGS on Wheat599) and a higher training cost on Wheat2000 and SoyBase; millisecond-scale inference is the operational advantage after a model has been trained.

The remainder of this paper is organized as follows. Section 2 details the BiMSGP architecture, evaluation metrics, and experimental setup. Section 3 presents the main experimental results, ablation studies, and computational efficiency analysis. Section 4 discusses the key findings, limitations, and implications. Finally, Section 5 concludes the paper and outlines future research directions.

## 2. Materials and Methods

This section details the datasets, data preprocessing procedures, model architecture, implementation details, and evaluation framework used in the development and assessment of the proposed BiMSGP model.

### 2.1. Datasets and Preprocessing

BiMSGP was evaluated on three publicly available genomic datasets (Table 1):

- **Wheat599**: 599 wheat lines genotyped with 1279 Diversity Arrays Technology (DArT) markers after quality control and grain yield (GY) measured in four environments [6,29].
- **Wheat2000**: 2000 Iranian bread wheat lines with 33,709 DArT markers and eight quantitative traits (Grain Length (GL), Grain Width (GW), Grain Hardness (GH), Thousand Kernel Weight (TKW), Test Weight (TW), Sodium Dodecyl Sulphate Sedimentation (SDS), Grain Protein (GP), and Plant Height (PHT)) [18,30].
- **SoyBase**: a soybean panel derived from the public SoyDNGP/SoyBase resources [20,31]. Gao et al. reported 13,784 accessions and 32,032 SNPs after their complete-case and marker-intersection filters. The processed genotype table used in this study contained 15,878 genotyped accessions and 42,195 SNP columns; we did not restrict the main tables to that 13,784 × 32,032 subset. After excluding accessions with missing values of the target phenotype, sample sizes ranged from 14,485 (Yield) to 15,878 (oil and protein). Seven quantitative traits (Plant Height (Hgt), Oil Content (Oil), Protein Content (Protein), Hundred-seed Weight (SdWgt), Flowering Date (R1), Maturity Date (R8), Yield) and four categorical traits (Flower Color (FC), Pubescence Density (P_DENS), Pod Color (POD), Stem Termination (ST)) were analyzed.

SNP markers (SoyBase) were encoded as {0, 1, 2}, representing the number of minor-allele copies. DArT markers in Wheat599 and Wheat2000 were encoded as binary values (0 = absence, 1 = presence). No additional marker selection was applied beyond the source quality-control procedures described below. Within each training fold, marker values were standardized with scikit-learn (v1.4.2) StandardScaler fitted on the training partition only and applied to the held-out partition (bimsgp/train.py). Phenotypes for regression were likewise standardized to zero mean and unit variance within each training fold; metrics were reported after inverse transformation to the original scale.

The original Wheat599 panel contained 1447 DArT markers. During preparation of the public dataset, markers with MAF < 0.05 were excluded, leaving 1279 markers. Missing genotypes were filled by marker-specific Bernoulli sampling, with probabilities estimated from the observed genotypes [29,32]. Both the main Wheat599 experiments and the ablation study used this same preprocessed matrix. We performed no additional genotype imputation for Wheat599 in this study. Wheat2000 retained 33,709 DArT markers after the quality control of Ma et al. [18]. For SoyBase, genotypes were read from the processed marker table (geno-big.parquet; 15,878 rows and 42,195 SNP columns) without applying the 32,032-SNP intersection or 13,784-accession complete-case filter of Gao et al. Samples with missing values of the target phenotype were then excluded, giving the trait-specific *n* in Table 1. No additional genotype imputation was applied in the training scripts. The public code expects these already-processed matrices (Geno.csv/wheat_GS_v2.RData/geno-big.parquet); scripts that rebuild the 1279-marker Wheat599 table or the 42,195-column SoyBase table from raw public downloads are not included in the released tree.

**Figure 1:**
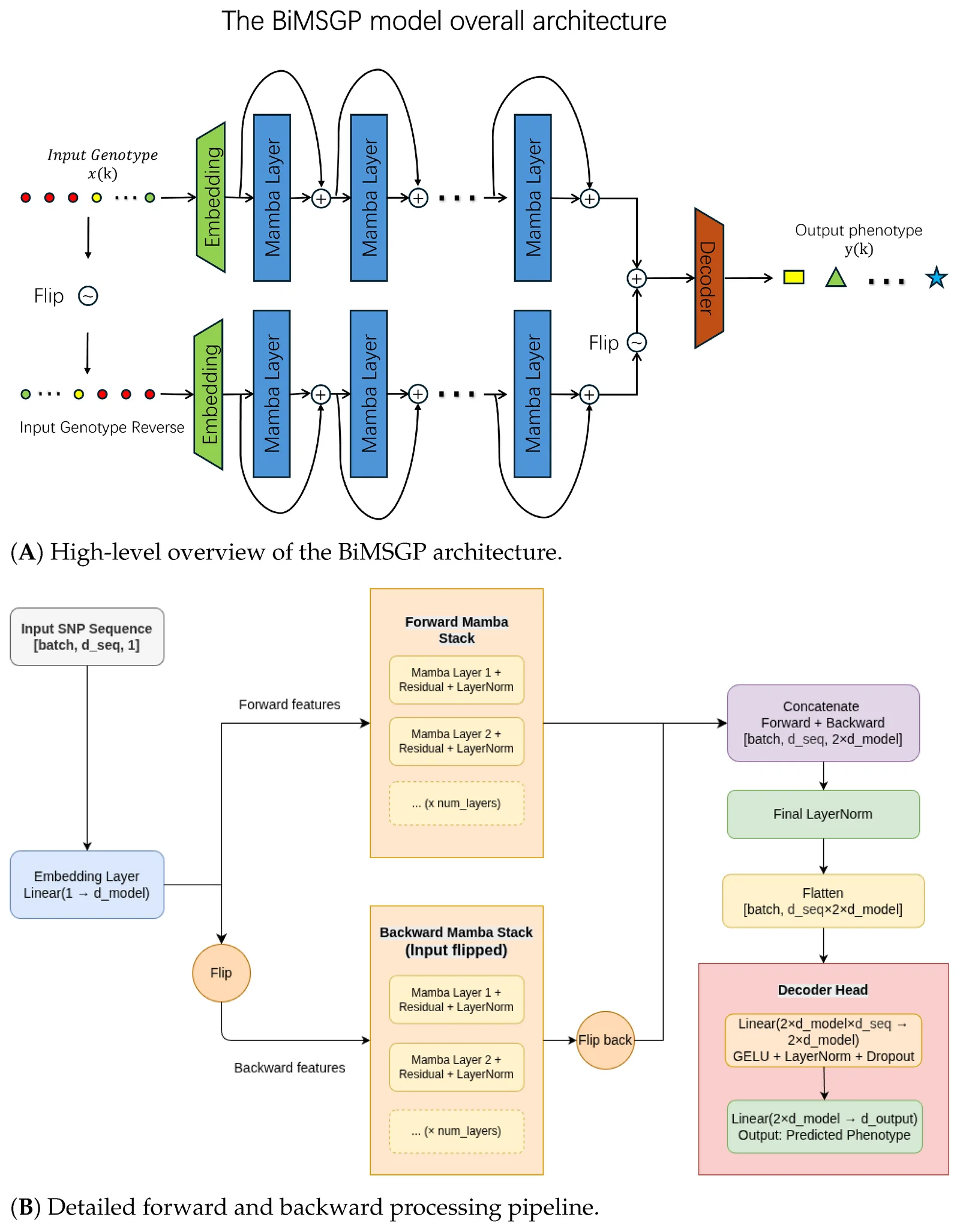
Bidirectional Mamba-based architecture of the proposed BiMSGP model. (**A**) High-level view showing the bidirectional design with embedding and decoder. Colored dots are schematic markers in source-file column order (not chromosome identities); the tilde denotes sequence reversal; plus signs denote residual addition; green parallelograms are embedding layers; blue bars are Mamba layers; the orange parallelogram is the decoder; the yellow rectangle, green triangle, and blue star are schematic trait outputs (regression or classification), not quantitative results. (**B**) Detailed implementation: gray, input marker tensor; light blue, embedding; orange, forward and backward Mamba stacks; orange circles, flip operations; purple, concatenation of the two directions; green, layer normalization; yellow, flattening; red, decoder head. Black arrows indicate data flow. The input is an abstract one-dimensional marker sequence in source-file column order; the schematic does not mark chromosome boundaries, and inter-chromosome (or otherwise unsorted) junctions are serialization artifacts rather than biological adjacency.

Markers were arranged as a one-dimensional sequence in the column order of the source genotype matrices. Wheat599 columns are DArT identifiers (for example, wPt and c names) and do not include chromosome or physical-position annotation, so the sequence is not guaranteed to follow genomic coordinates. Wheat2000 followed the column order of the genotype matrix in the public wheat_GS_v2.RData object. SoyBase SNPs followed the column order of the processed genotype table. Concatenating markers across chromosomes, or using an unsorted marker list, places the last marker of one group next to the first marker of the next; such junctions are artifacts of serialization rather than biological adjacency. Figure 1 is an abstract schematic of this marker sequence and does not depict chromosome boundaries.

Class counts and proportions for the four SoyBase categorical traits are given in Table 2. Stem termination (ST) is the most imbalanced (class S: 8.1%), which is why balanced accuracy is reported alongside overall accuracy.

All datasets are publicly accessible from their original publications or repositories [6,18,20].

### 2.2. BiMSGP Model Architecture

The proposed BiMSGP model adopts a bidirectional selective state space modeling approach to effectively capture long-range dependencies among genomic markers while maintaining computational efficiency.

#### 2.2.1. Background on Mamba Architecture

The Mamba model [27] is a recent state space model (SSM) designed as an efficient alternative to Transformers for modeling long sequences. Unlike Transformers, which suffer from quadratic computational complexity with respect to sequence length, Mamba achieves linear time and memory complexity through a selective state space mechanism.

Specifically, Mamba is based on a structured state space model (S4) [26] with the following key innovations:

- **Selective State Space**: In the selective state-space formulation, the learned state-transition matrix *A* is input-independent, while input-dependent projections generate *B*, *C*, and the discretization step size Δ. This selectivity allows the network to dynamically focus on relevant parts of the sequence while filtering out noise, which is useful for high-dimensional genomic marker data.
- **Hardware-aware Algorithm**: Mamba employs a parallel scan algorithm that enables efficient GPU-parallel computation, significantly improving training and inference speed compared to standard SSMs.
- **Linear Complexity**: For a sequence of *M* markers, Mamba has 𝒪(*M*) time and memory complexity, making it particularly suitable for genomic data with tens of thousands of markers (M=1279–42,195 in our experiments).

In standard Mamba, information flows only in one direction (forward). To better capture long-range bidirectional dependencies among SNP markers, we propose **BiMSGP**, which extends Mamba into a bidirectional architecture.

#### 2.2.2. Overall Framework

As illustrated in Figure 1A, BiMSGP consists of an embedding layer, bidirectional Mamba stacks, and a decoder head. The marker sequence is embedded, processed in the forward and reverse directions, concatenated, and decoded to a trait prediction. Figure 1B shows the same pipeline in more detail: a linear embedding maps each marker to $d_{\mathrm{model}}$, residual Mamba blocks (when used) run on the original and flipped sequences, the two representations are concatenated, and a lightweight MLP decoder produces the output. Chromosome boundaries are not marked; the figure is an abstract marker sequence in source-file column order.

#### 2.2.3. Input and Embedding Layer

The input to BiMSGP is a matrix of genomic markers with shape [B,M,1], where *B* is the batch size and *M* is the number of markers (sequence length). Each genomic marker is initially treated as a univariate feature.

A linear embedding layer projects the input from 1 dimension to the model dimension $d_{\mathrm{model}}$:

(1) $$X_{\mathrm{emb}}=\mathrm{Linear}\left(1\to d_{\mathrm{model}}\right)\cdot X_{\mathrm{input}}$$

where $X_{\mathrm{input}}$ denotes the input marker tensor of shape [B,M,1]. This produces an embedded representation $X_{\mathrm{emb}}$ of shape $\left[B,M,d_{\mathrm{model}}\right]$ that serves as input to the subsequent bidirectional Mamba stacks. The embedding layer allows the model to learn a richer representation of the genomic markers before processing them through the state space layers.

#### 2.2.4. Bidirectional Mamba Stacks

The core of BiMSGP is the bidirectional Mamba architecture, which processes the marker sequence in both forward and backward directions to capture long-range dependencies from both ends.

For the **forward path**, the sequence passes through a stack of Mamba layers with residual connections and layer normalization:

(2) $$X_{\mathrm{fwd}}^{\left(l\right)}=\mathrm{LayerNorm}\left(X_{\mathrm{fwd}}^{\left(l-1\right)}+\mathrm{Mamba}\left(X_{\mathrm{fwd}}^{\left(l-1\right)}\right)\right),\;\;\;l=1,\ldots ,L$$

where *L* is the number of layers in the stack. In our experiments, we typically set *L* to 1 or 2, as deeper stacks did not yield further improvements according to the ablation studies.

For the **backward path**, the sequence is first flipped, processed by an identical Mamba stack, and then flipped back:

(3) $$X_{\mathrm{bwd}}=\mathrm{Flip}\left(\mathrm{MambaStack}\left(\mathrm{Flip}\left(X_{\mathrm{emb}}\right)\right)\right)$$

In configurations with residual connections, a residual connection and layer normalization are applied after each Mamba layer to stabilize training; the ablation study also evaluates configurations without residual connections.

#### 2.2.5. Feature Fusion and Decoder Head

The forward and backward features are concatenated along the feature dimension:

(4) $$X_{\mathrm{fused}}=\mathrm{Concat}\left(\left[X_{\mathrm{fwd}},X_{\mathrm{bwd}}\right]\right)\in R^{B\times M\times 2d_{\mathrm{model}}}$$

After a final layer normalization, the fused representation is flattened and passed through a lightweight decoder head:

(5) $$\hat{y}=\mathrm{Decoder}\left(\mathrm{Flatten}(X_{\mathrm{fused}})\right)$$

Two decoder heads are used (bimsgp/model.py). Wheat599 and Wheat2000 use the wide head (three linear layers; first dropout at least 0.15). SoyBase uses the compact head (two linear layers; dropout 0.1). Let $d_{\mathrm{in}}=2d_{\mathrm{model}}M$ for bidirectional models.

The Wheat (wide) decoder is

(6) $$\begin{array}{cc} & \mathrm{Linear}\left(d_{\mathrm{in}}\to 4d_{\mathrm{model}}\right)\to \mathrm{GELU}\to \mathrm{LayerNorm}\to \mathrm{Dropout}\left(0.15\right)\\ & \;\to \mathrm{Linear}\left(4d_{\mathrm{model}}\to 2d_{\mathrm{model}}\right)\to \mathrm{GELU}\to \mathrm{Dropout}\left(0.1\right)\\ & \;\to \mathrm{Linear}(2d_{\mathrm{model}}\to d_{\mathrm{output}}). \end{array}$$

The SoyBase (compact) decoder is

(7) $$\begin{array}{cc} & \mathrm{Linear}\left(d_{\mathrm{in}}\to 2d_{\mathrm{model}}\right)\to \mathrm{GELU}\to \mathrm{LayerNorm}\to \mathrm{Dropout}\left(0.1\right)\\ & \;\to \mathrm{Linear}(2d_{\mathrm{model}}\to d_{\mathrm{output}}). \end{array}$$

Here, $d_{\mathrm{output}}$ denotes the task-specific output dimension: one output is used for regression, whereas classification uses the configured number of output logits. This design enables BiMSGP to effectively model complex non-linear marker–trait associations from both directions while keeping the model computationally tractable.

#### 2.2.6. Implementation Details

The model was implemented in PyTorch (v2.2.2) using the official mamba-ssm library (v1.2.2). All experiments were conducted on Ubuntu Linux (v22.04.5 LTS) systems with NVIDIA GPUs, including A100 GPUs with 40 GB or 80 GB of memory, an RTX 3090, and an RTX 4090 (NVIDIA Corporation, Santa Clara, CA, USA).

The default main-table configuration (Table 3, Table 4, Table 5 and Table 6) used BiMSGP with $d_{\mathrm{model}}=64$, $d_{\mathrm{state}}=64$, L=1 bidirectional Mamba layer per direction, residual connections and layer normalization after each Mamba block, AdamW (learning rate $5\times 10^{-4}$, weight decay 0.01), ReduceLROnPlateau (patience 10, factor 0.5), and a maximum of 100 epochs. Wheat599 (Table 3) and Wheat2000 (Table 4) used the three-layer wide decoder (GELU; dropout 0.15 then 0.1) and batch size 8. SoyBase quantitative traits (Table 5) used the two-layer compact decoder (GELU; dropout 0.1) and batch size 32, except oil and R8, which used L=2 and 20 epochs rather than the L=1/100-epoch default. SoyBase categorical traits (Table 6) used the compact decoder and batch size 1. The Wheat599 residual-on L=1 setting in Table 3 has 42.0 M parameters (Environment 1 mean PCC 0.6198); residual-off and other depth/dimension variants are reported only in the ablation subsection. Published table numbers were obtained from internal mixed-precision (FP16 autocast) runs. The public trainer (https://github.com/liuxumail-create/BiMSGP, accessed on 17 September 2026; bimsgp/train.py) defaults to FP32 and provides –amp to request the original mixed-precision setting; we have not re-tabulated the published means under the FP32 default. For regression, continuous phenotypes were standardized to zero mean and unit variance within each training fold.

### 2.3. Competing Methods

To evaluate BiMSGP, we compared it against four widely used genomic prediction methods: GBLUP implemented with BGLR (v1.1.4), DeepGS (v1.2), DNNGP (v3.1), and SoyDNGP (v0.1.2). Brief descriptions of each method are provided below.

#### 2.3.1. GBLUP

Genomic best linear unbiased prediction (GBLUP) is one of the most widely adopted linear mixed model approaches for genomic selection [11,12]. It replaces the traditional pedigree-based relationship matrix with a genomic relationship matrix (GRM) constructed from genome-wide markers. The model can be expressed as:

(8) y=Xβ+Zg+ϵ

where y is the vector of phenotypes, β represents fixed effects, g is the vector of genomic breeding values with $g∼N(0,G\sigma_{g}^{2})$, G is the GRM, and ϵ is the residual error. GBLUP assumes additive genetic effects and has low computational cost, making it a strong baseline for genomic prediction across various species.

#### 2.3.2. DeepGS

DeepGS is a deep learning-based method for genomic prediction that utilizes a deep convolutional neural network (CNN) to extract hidden features directly from raw genotypic data [18]. The model treats SNP markers as a one-dimensional sequence and applies convolutional layers, pooling, and fully connected layers to learn complex non-linear patterns. DeepGS was one of the early attempts to apply deep learning to genomic selection and has demonstrated improved performance over traditional linear models on several crop datasets by capturing non-linear and non-additive marker effects.

#### 2.3.3. DNNGP

Deep Neural Network Genomic Prediction (DNNGP) is a deep learning framework specifically designed for integrating multi-omics data in plant genomic prediction [6]. It employs a hierarchical multilayer neural network with batch normalization, ReLU activation, and early stopping to mitigate overfitting. DNNGP can handle various types of omics data and dynamically learns feature representations from raw inputs. It has shown competitive or superior performance compared to GBLUP and other deep learning methods, particularly on large-scale breeding datasets, while maintaining reasonable computational efficiency.

#### 2.3.4. SoyDNGP

SoyDNGP is a soybean-specific deep learning framework for genomic prediction [20]. It converts standard VCF files into 3D tensor inputs and employs a convolutional neural network (CNN) architecture optimized for soybean breeding traits. The model is designed to be web-accessible and user-friendly, focusing on capturing complex patterns in high-dimensional SNP data for traits such as yield, oil content, and protein content. SoyDNGP has demonstrated strong performance on large soybean panels and serves as a specialized competitor for legume genomic prediction tasks.

### 2.4. Evaluation Metrics

To rigorously assess the predictive performance of BiMSGP and competing methods, we employed multiple complementary evaluation metrics suitable for both regression and classification tasks in genomic prediction.

For all **quantitative traits (regression tasks)**, the primary metric was the **Pearson correlation coefficient (PCC)** between predicted and observed phenotypic values, which measures the strength of the linear relationship and is widely used in genomic selection studies because it is invariant to scale. We also reported the **root mean squared error (RMSE)** and **mean absolute error (MAE)** to evaluate the accuracy of absolute predictions:

(9) $$\mathrm{PCC}=\frac{\sum_{i=1}^{n}\left(y_{i}-\bar{y}\right)\left({\hat{y}}_{i}-\bar{\hat{y}}\right)}{\sqrt{\sum_{i=1}^{n}{\left(y_{i}-\bar{y}\right)}^{2}\sum_{i=1}^{n}{\left({\hat{y}}_{i}-\bar{\hat{y}}\right)}^{2}}}$$

(10) $$\mathrm{RMSE}=\sqrt{\frac{1}{n}\sum_{i=1}^{n}{\left(y_{i}-{\hat{y}}_{i}\right)}^{2}},\;\;\;\mathrm{MAE}=\frac{1}{n}\sum_{i=1}^{n}\left|y_{i}-{\hat{y}}_{i}\right|$$

where *n* is the number of evaluated samples, $y_{i}$ and ${\hat{y}}_{i}$ are the observed and predicted values, respectively, and $\bar{y}$, $\bar{\hat{y}}$ denote their means.

All regression metrics were computed on the original (unstandardized) phenotypic scale after inverse transformation.

For the four **categorical traits** in the SoyBase dataset (flower color (FC), pubescence density (P_DENS), pod color (POD), and stem termination (ST)), we used **classification accuracy** and **balanced accuracy** as the main metrics. Accuracy is the proportion of correctly classified samples:

(11) $$\mathrm{Accuracy}=\frac{1}{n}\sum_{i=1}^{n}1\left({\hat{y}}_{i}=y_{i}\right),$$

where 1(·) is the indicator function. Balanced accuracy is particularly suitable for potentially imbalanced classes and is calculated as the average of recall across all classes:

(12) $$\mathrm{Balanced}\;\mathrm{Accuracy}=\frac{1}{C}\sum_{c=1}^{C}\frac{{\mathrm{TP}}_{c}}{{\mathrm{TP}}_{c}+{\mathrm{FN}}_{c}}$$

where *C* is the number of classes and ${\mathrm{TP}}_{c}$ and ${\mathrm{FN}}_{c}$ are the true positives and false negatives for class *c*, respectively.

All performance metrics were evaluated using 10-fold cross-validation. Regression used scikit-learn KFold (10 splits, shuffle, random_state = 42). SoyBase classification used StratifiedKFold with the same split count and seed. Within each task, BiMSGP and the baseline methods shared those fold assignments so that methods could be compared on identical held-out partitions. Ten-fold cross-validation was performed without a separate validation subset within each training fold. For neural-network models, the held-out fold was used both to select the epoch/checkpoint (highest PCC for regression; highest balanced accuracy for classification) and to calculate the reported performance metrics. Held-out performance also guided learning-rate adjustment: ReduceLROnPlateau was stepped on held-out RMSE (regression) or 1−balanced accuracy (classification). Consequently, the reported scores are subject to potential model-selection bias and should not be interpreted as unbiased estimates of generalization performance. Architectural and initial optimizer settings in Implementation Details were kept fixed across folds for each dataset or trait; no fold-specific hyperparameter search was performed. Those fixed settings do not remove the selection bias from checkpointing and learning-rate updates on the held-out fold. GBLUP has no epoch-wise checkpointing and is not subject to the same selection step. The extent to which this model-selection bias affects comparisons between methods cannot be determined under the current evaluation protocol, and relative differences between methods may also be influenced by this validation procedure.

Paired comparisons used the ten shared folds and the fold-wise PCC of BiMSGP and each baseline (Appendix A Table A1, Table A2 and Table A3). Let $\Delta_{i}$ be the PCC difference on fold *i* (BiMSGP minus the comparator) and $\bar{\Delta }$ its mean. We report $\bar{\Delta }$, a Student’s *t* 95% confidence interval for $\bar{\Delta }$ with 9 degrees of freedom, and Cohen’s paired effect size

(13) $$d_{z}=\frac{\bar{\Delta }}{s_{\Delta }},$$

where $s_{\Delta }$ is the sample standard deviation of the ten $\Delta_{i}$. The primary test is a two-sided paired *t*-test on $\left\{\Delta_{i}\right\}$; a two-sided Wilcoxon signed-rank test is reported as a supplementary check of the same differences. Shared 10-fold CV makes the comparisons paired, but the training partitions overlap across folds, so the $\Delta_{i}$ are not independent experiments. Ordinary paired *t* and Wilcoxon procedures can therefore understate uncertainty, and Wilcoxon still assumes exchangeable paired observations. We treat both *p*-values as exploratory. They are unadjusted for the number of trait-by-method contrasts; Holm or other multiplicity correction was not applied. RMSE and SoyBase classification accuracy/balanced accuracy were not subjected to these tests and are interpreted only as numerical comparisons. Fold-level scores were not archived for Wheat599 SoyDNGP or for SoyBase SoyDNGP, so those comparisons remain descriptive (mean ± SD only). For each trait and metric, boldface in the main tables indicates the numerically best mean across models and does not indicate statistical significance.

## 3. Results

**Figure A1:**
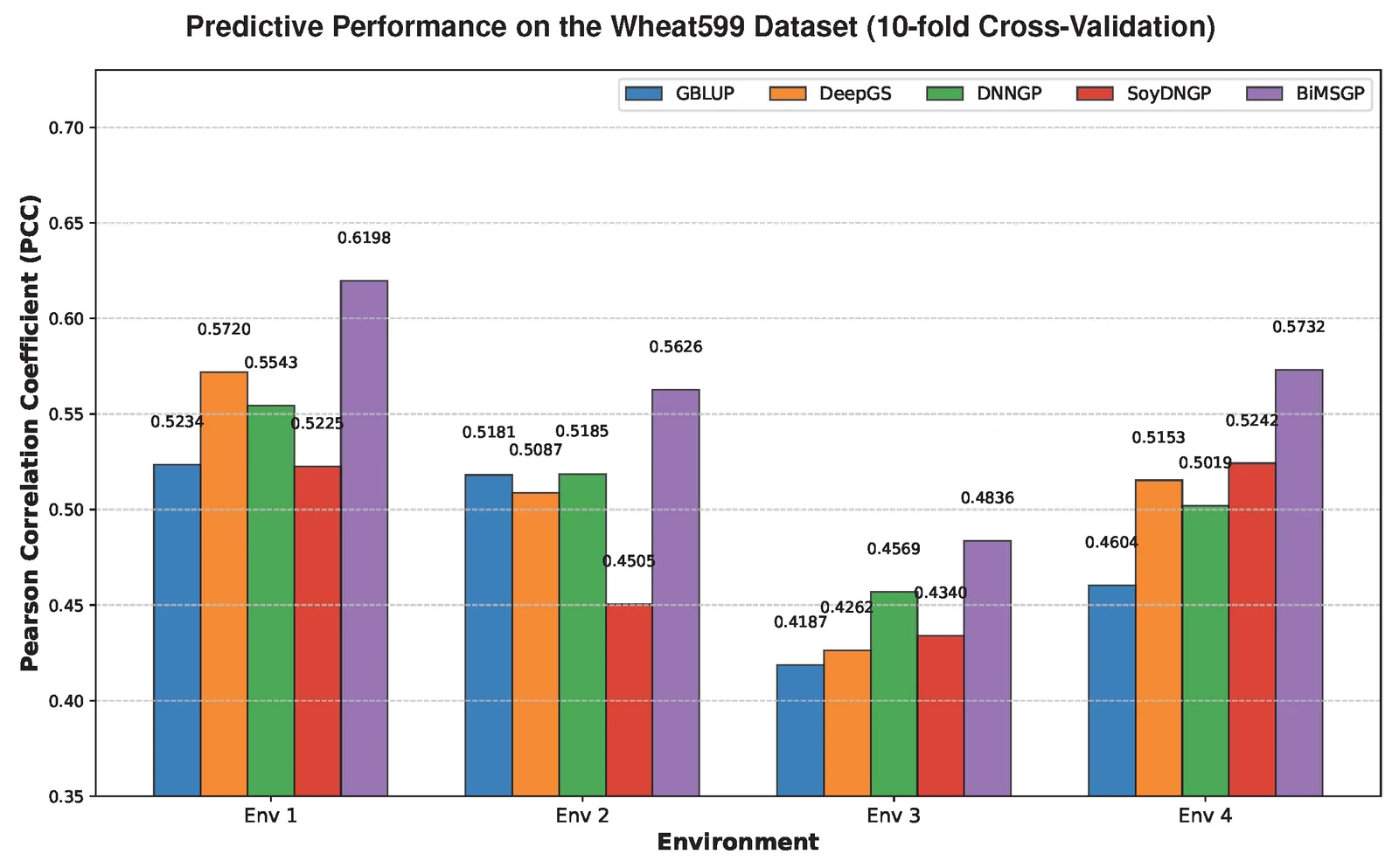
Graphical summary of Table 3. Mean PCC on Wheat599 across four environments (10 folds). For each environment, bar height is the numerical mean and does not indicate statistical significance.

**Figure A2:**
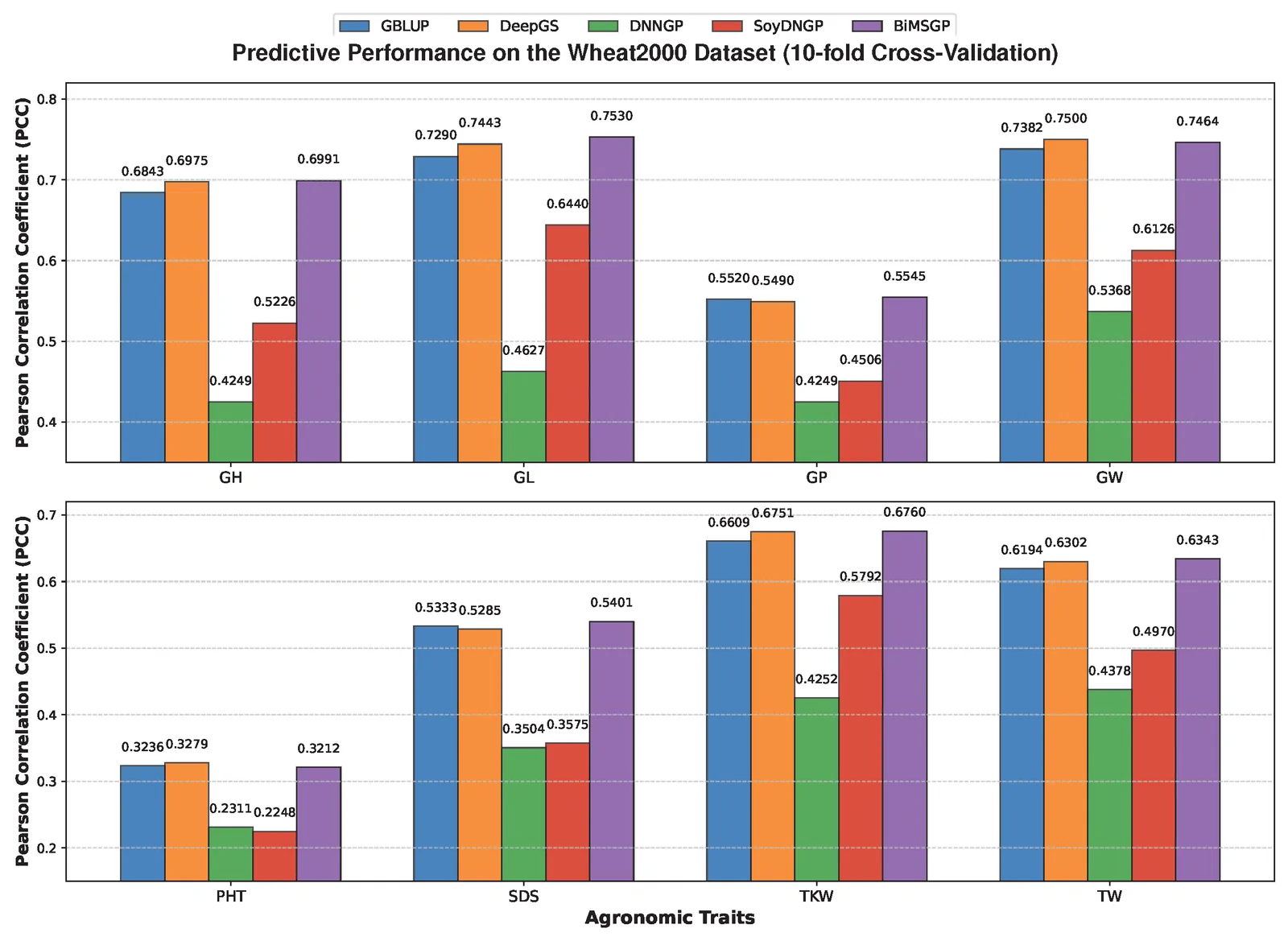
Graphical summary of Table 4. Mean PCC on Wheat2000 across eight traits (10 folds). For each trait, bar height is the numerical mean and does not indicate statistical significance.

**Figure A3:**
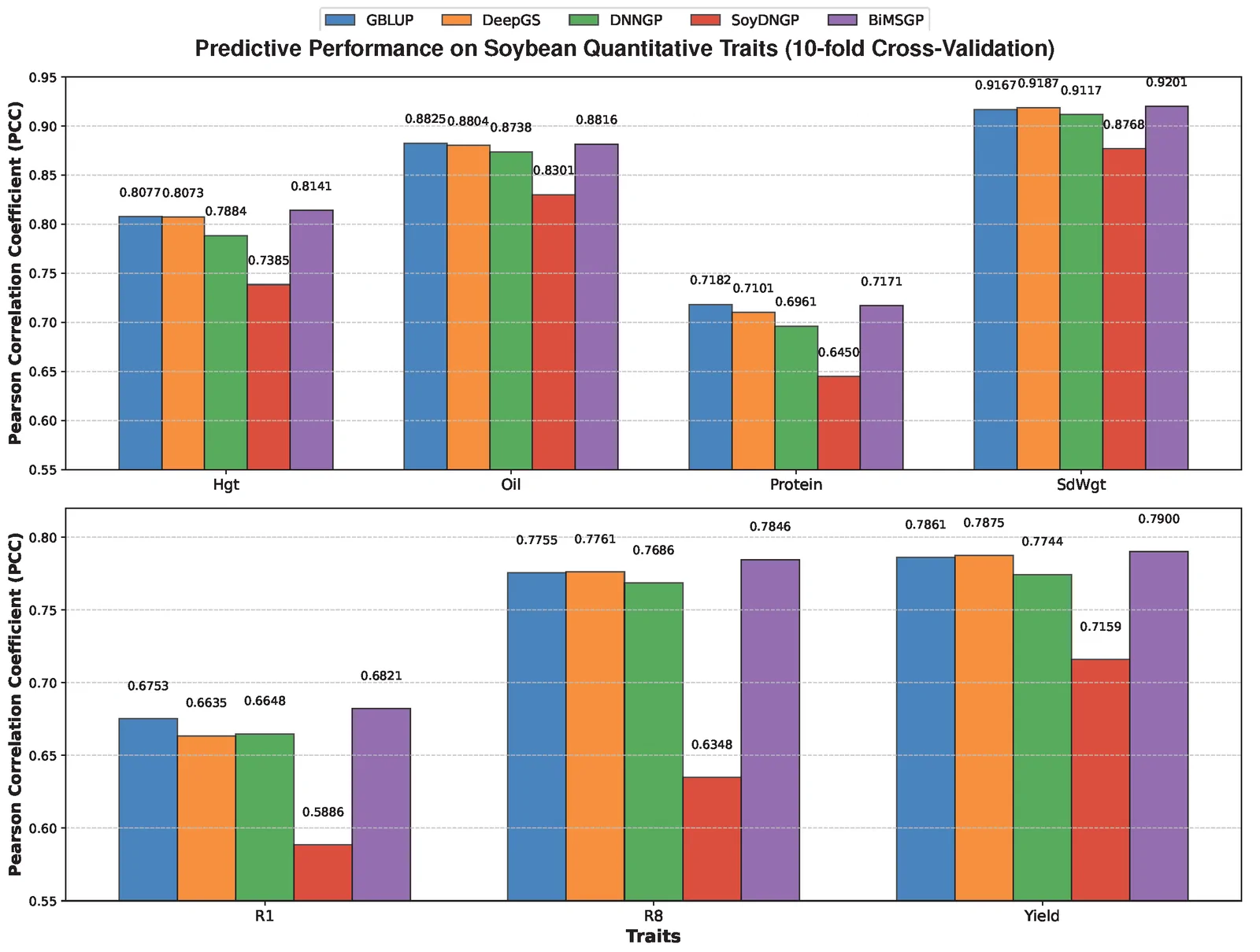
Graphical summary of Table 5. Mean PCC on soybean quantitative traits (10 folds). (**Upper panel**): first four traits; (**lower panel**): remaining three. For each trait, bar height is the numerical mean and does not indicate statistical significance.

**Figure A4:**
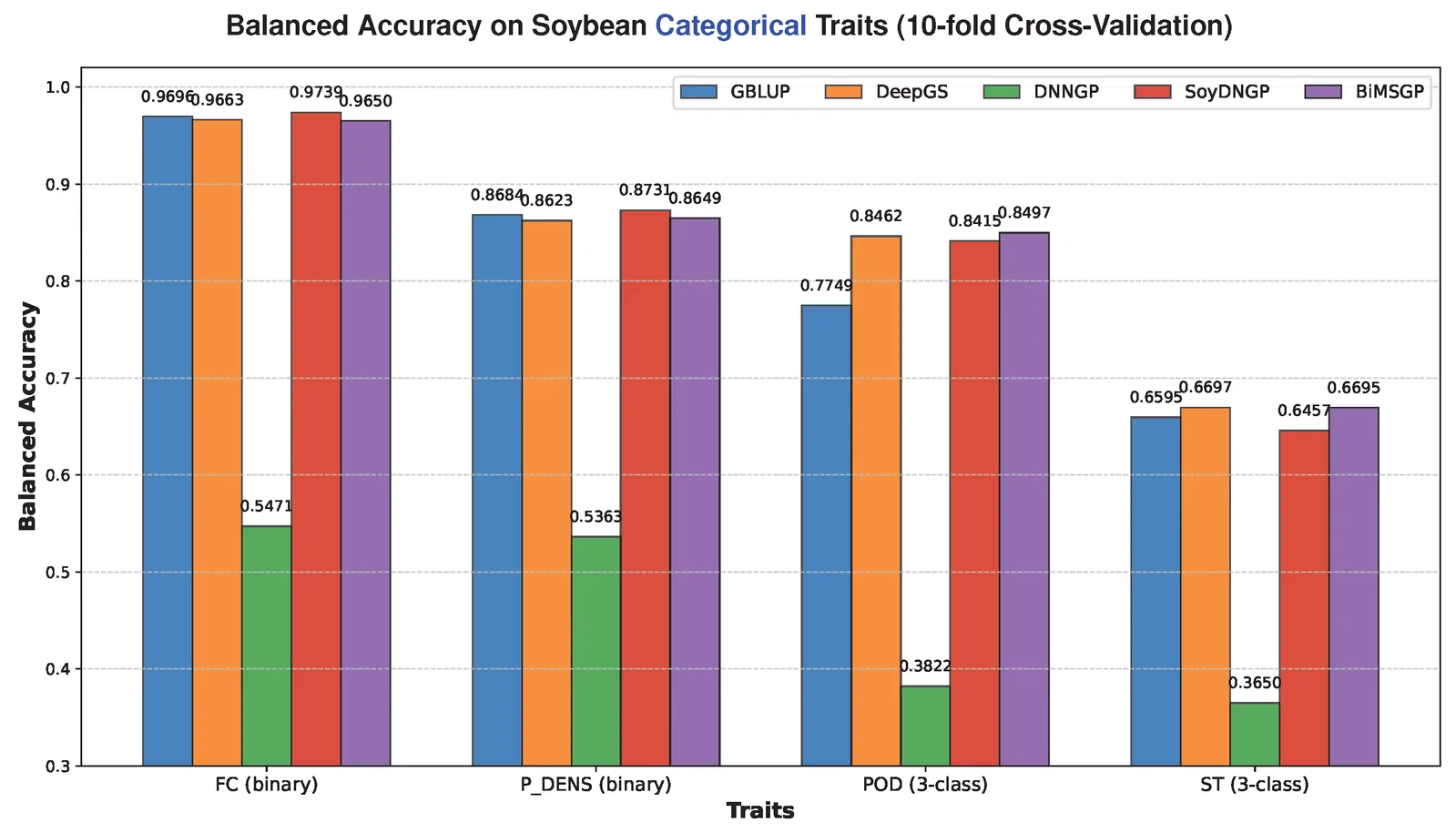
Graphical summary of Table 6. Mean balanced accuracy on soybean categorical traits (10 folds). For each trait, bar height is the numerical mean and does not indicate statistical significance. Balanced accuracy was not included in the paired PCC tests.

In this section, we evaluate the proposed BiMSGP model against GBLUP [12], DeepGS [18], DNNGP [6], and SoyDNGP [20] on Wheat599 [6], Wheat2000 [18,30], and SoyBase [20,31]. All three datasets were used for regression, whereas classification was evaluated only on SoyBase; all evaluations used 10-fold cross-validation with shared fold assignments. For neural-network models, the held-out fold was also used for checkpoint selection and learning-rate adjustment, so the reported means are exploratory under this protocol and may be optimistic. We distinguish numerically highest mean PCC from exploratory paired tests of fold-wise PCC differences (Appendix A Table A1, Table A2 and Table A3); those tests do not remove selection bias. RMSE and classification metrics are numerical comparisons only. Bar-plot summaries of the primary metric are in Appendix C (Figure A1, Figure A2, Figure A3 and Figure A4) and are not repeated in the main text. We then report ablation studies and computational cost.

### 3.1. Performance on Wheat599 Dataset

Wheat599 comprises 599 lines and 1279 DArT markers in four mega-environments [6]. Phenotypes were standardized per environment; PCC is the primary metric and RMSE the secondary metric.

BiMSGP had the numerically highest mean PCC in all four environments (Table 3). In Environment 1, the mean PCC was 0.6198 ± 0.0708 (RMSE 0.8038 ± 0.0591), compared with 0.5720 ± 0.0763 for DeepGS, 0.5543 ± 0.1015 for DNNGP, 0.5234 ± 0.0798 for GBLUP, and 0.5225 ± 0.1209 for SoyDNGP. Exploratory paired *t*-tests on fold-wise PCC (Table A1) gave $p_{t}\leq 0.006$ versus GBLUP in all four environments; versus DeepGS, $p_{t}=0.039$ and 0.027 in Environments 1 and 3, and $p_{t}=0.083$ in Environments 2 and 4; and versus DNNGP, $p_{t}<0.05$ in Environments 1, 2, and 4, but not Environment 3 ($p_{t}=0.141$). These $p_{t}$ values are unadjusted and do not account for overlapping CV folds. RMSE is reported numerically and was not tested. SoyDNGP fold-level scores were not archived for Wheat599, so that comparison remains descriptive.

### 3.2. Performance on Wheat2000 Dataset

Wheat2000 comprises 2000 Iranian bread wheat landraces and 33,709 DArT markers. Eight traits were analyzed: grain hardness (GH), grain length (GL), grain protein (GP), grain width (GW), plant height (PHT), SDS sedimentation (SDS), thousand kernel weight (TKW), and test weight (TW).

As shown in Table 4, BiMSGP had the numerically highest mean PCC for six of eight traits (GH, GL, GP, SDS, TKW, and TW). All eight traits were tested against each baseline on the shared folds (Table A2). Versus DeepGS, none of those six fold-wise PCC differences had exploratory $p_{t}<0.05$ (smallest $p_{t}=0.093$ for GL; TKW ΔPCC =+0.0008, $p_{t}=0.837$). DeepGS had a higher mean PCC for grain width (0.7500 ± 0.0331 versus 0.7464 ± 0.0384) and plant height (0.3279 ± 0.0617 versus 0.3212 ± 0.0674), with $p_{t}=0.325$ and 0.473. A non-significant $p_{t}$ does not imply that the two models are equivalent. Versus GBLUP, exploratory $p_{t}<0.05$ for GH, GL, GW, TKW, and TW, but not for GP, SDS, or PHT. Versus DNNGP and SoyDNGP, $p_{t}<0.05$ for all eight traits. RMSE was not tested.

### 3.3. Performance on Soybean Dataset (SoyBase)

We further evaluated BiMSGP on the larger SoyBase panel using the same within-dataset 10-fold protocol (trait-specific *n* of 14,485–15,878 and 42,195 SNPs in the processed matrix). Seven quantitative traits (plant height (Hgt), oil content (Oil), protein content (Protein), hundred-seed weight (SdWgt), flowering date (R1), maturity date (R8), and yield (Yield)) and four categorical traits (flower color (FC), pubescence density (P_DENS), pod color (POD), and stem termination (ST)) were analyzed. For quantitative traits, performance was measured by PCC and RMSE; for classification, accuracy and balanced accuracy were reported.

As shown in Table 5, BiMSGP had the numerically highest mean PCC on five of seven quantitative traits: Hgt (0.8141 ± 0.0135), SdWgt (0.9201 ± 0.0073), R1 (0.6821 ± 0.0114), R8 (0.7846 ± 0.0176), and yield (0.7900 ± 0.0093). GBLUP had a higher mean PCC for oil (0.8825 versus 0.8816) and protein (0.7182 versus 0.7171). Exploratory paired tests of fold-wise PCC (Table A3) gave $p_{t}=0.025$ in favour of GBLUP for oil (ΔPCC =−0.0010) and $p_{t}=0.706$ for protein. Versus GBLUP, $p_{t}<0.05$ for Hgt, SdWgt, R1, and R8; yield had $p_{t}=0.074$ versus GBLUP and 0.193 versus DeepGS. Versus DeepGS, $p_{t}<0.05$ only for Hgt, R1, and R8. RMSE was not tested.

For the classification tasks (Table 6), BiMSGP was competitive but was not the top method on every metric. SoyDNGP attained the highest accuracy and balanced accuracy on the binary traits FC and P_DENS, while DeepGS performed best on the three-class trait ST. BiMSGP had the highest balanced accuracy on POD (0.8497 ± 0.0123). Class proportions (Table 2) show substantial imbalance, especially for ST (class S: 8.1%), which is consistent with the gap between overall accuracy (0.8179) and balanced accuracy (0.6695) for that trait. Fold-level classification scores were not archived for all methods, so these ranks are descriptive.

Overall, under the reported evaluation protocol, BiMSGP obtained competitive mean scores for both regression and classification. Numerical rank should not be read as an unbiased or uniform advantage, particularly for SoyBase oil and protein and for most Wheat2000 traits versus DeepGS. Classification ranks in Table 6 are numerical only.

### 3.4. Ablation Studies on Model Architecture

To systematically evaluate the contribution of key components in the proposed Mamba-based genomic prediction framework, we performed a comprehensive set of ablation experiments on the Wheat599 dataset. The ablation study used the same preprocessed Wheat599 matrix containing 1279 markers as the main Wheat599 experiments, as described in Section 2.1 Phenotypic values for Environment 1 were standardized to zero mean and unit variance (mean ± standard deviation: 0.0000 ± 0.9992). All ablation configurations were trained under identical optimization settings: learning rate 0.0005, weight decay 0.01, 100 epochs, and batch size 16. Performance was assessed via 10-fold cross-validation using Pearson correlation coefficient (PCC, higher is better), root mean squared error (RMSE), and mean absolute error (MAE).

The ablation study examined five main factors: (i) presence/absence of residual connections, (ii) number of layers (*L*), (iii) model dimension ($d_{\mathrm{model}}$), (iv) state dimension ($d_{\mathrm{state}}$), and (v) architectural variants (standard bidirectional Mamba vs. forward-only, backward-only, and sum-pooling variants).

Table 7 summarizes the computational cost of different configurations. Trainable parameter counts ranged from approximately 21 M (smallest model) to 673 M (largest), while average training time for 100 epochs varied from 0.96 min (lightweight forward Mamba) to 8.28 min (large $d_{\mathrm{model}}=256$ model). Inference time per sample remained efficient (1.64–1.98 ms) across all configurations.

Table 8 reports residual-on versus residual-off stacks at several depths on Wheat599 Environment 1 (fixed $d_{\mathrm{model}}=64$, $d_{\mathrm{state}}=64$). The main Wheat599 comparison in Table 3 used residual-on L=1 with batch size 8 and mean PCC 0.6198, whereas the ablation runs used batch size 16; the residual-on L=1 ablation row therefore reports 0.6191. On this ablation panel, residual-off variants had a higher mean PCC at every tested depth, with no increase in RMSE or MAE. Mean PCC was highest for shallow architectures (*L* = 1–4); increasing depth to L=10 produced a slight decline. These are numerical comparisons on this dataset and environment, not a claim that residual connections reduce predictive performance in general.

Table 9 isolates the impact of model dimension, state dimension, and directional variants (all with L=2). Increasing $d_{\mathrm{model}}$ from 32 to 128 improved PCC, with the peak at $d_{\mathrm{model}}=128$ (PCC 0.6387 ± 0.0835). Further scaling to $d_{\mathrm{model}}=256$ yielded comparable performance at substantially higher computational cost (parameters increased ∼4×, from 168 M to 673 M). The state dimension $d_{\mathrm{state}}=32$ performed marginally better than 16 or 64. Among directional variants, the standard bidirectional Mamba had a higher mean PCC than forward-only, backward-only, and sum-pooling configurations, indicating that bidirectional scanning is useful for using marker context from both directions.

For Wheat599 Environment 1, the main comparison in Table 3 used the residual-on configuration with L=1 and $d_{\mathrm{model}}=d_{\mathrm{state}}=64$, yielding a mean PCC of 0.6198. In the ablation study, the residual-off variant with L=1 achieved a higher mean PCC of 0.6387 (Table 8). This variant was evaluated separately and was not used in the main comparison tables. The higher mean PCC is an ablation finding for this dataset and environment and does not establish that removing residual connections is preferable across all datasets and traits. A similar mean PCC of 0.6387 also appeared for residual-on $d_{\mathrm{model}}=128$ at L=2 (Table 9); that setting was likewise not used in the main tables.

### 3.5. Computational Efficiency

We evaluated the computational efficiency of BiMSGP on the Wheat599 dataset (n=599, m=1279), which represents a typical small-to-medium-scale genomic prediction scenario. Training time is reported for 100 epochs for the neural-network models, whereas GBLUP time is reported per cross-validation fold.

Dashes for GBLUP indicate that it is not a neural network: there is no comparable trainable-parameter count, training is reported per cross-validation fold rather than per 100 epochs, and a separate per-sample neural inference time was not recorded. As shown in Table 10, BiMSGP used approximately 42 million parameters and 1.52 min for 100 epochs on Wheat599, compared with 81 K parameters and 0.40 min for DeepGS and 10.4 M parameters and 0.48 min for DNNGP. The Environment 1 mean PCC was numerically higher (0.6198 versus 0.5720 for DeepGS). Inference remained fast at 1.7 ms per sample.

Training times on Wheat2000 and SoyBase were much longer (Appendix B, Table A4): about 174 min per 100 epochs on Wheat2000 (1.105 billion parameters) and 462 min on SoyBase (691 million parameters), versus minutes for DeepGS. This training cost is a real practical constraint. Millisecond inference is the advantage after a model has been trained, not a claim that BiMSGP is cheaper to fit.

## 4. Discussion

The results indicate that BiMSGP is a competitive bidirectional Mamba-based model for genomic prediction under within-dataset 10-fold cross-validation. Model ranking depended on the crop, trait, and baseline, which is consistent with broader benchmarking evidence that no single algorithm is universally best [8,9,16,17]. Bidirectional scanning lets each marker use context from both directions and may help the model use long-range marker dependencies; the experiments do not demonstrate biological epistasis between distant loci.

On Wheat599, BiMSGP had the numerically highest mean PCC in all four environments, with the largest contrast versus DeepGS in Environment 4 (0.5732 versus 0.5153). Exploratory paired tests of fold-wise PCC gave $p_{t}\leq 0.006$ versus GBLUP in every environment; not every contrast with DeepGS or DNNGP had $p_{t}<0.05$. With n=599, fold-to-fold variation is large, so overlapping intervals should be interpreted cautiously.

On Wheat2000 (n=2000, m=33,709), BiMSGP had the numerically highest mean PCC on six of eight traits (GH, GL, GP, SDS, TKW, and TW). All eight traits were compared with DeepGS; none of those six differences had exploratory $p_{t}<0.05$, and DeepGS was numerically higher for GW and PHT. Versus GBLUP, $p_{t}<0.05$ for several grain-related traits, whereas GP, SDS, and PHT did not. These mean scores are consistent with applying the architecture to tens of thousands of markers under the reported protocol, but they do not establish unbiased generalization or uniform superiority over strong convolutional or additive baselines, and a non-significant $p_{t}$ does not imply equivalence.

On SoyBase, GBLUP remained a strong additive baseline. Exploratory $p_{t}=0.025$ favored GBLUP for oil and $p_{t}=0.706$ for protein. Versus GBLUP, $p_{t}<0.05$ for Hgt, SdWgt, R1, and R8; yield did not. Classification performance is reported as mean accuracy and balanced accuracy only and was competitive rather than uniformly best; ST in particular is imbalanced. These evaluations are within-dataset cross-validation, not prediction into an independent population, year, or environment.

Ablation studies on Wheat599 Environment 1 showed that bidirectional processing had a higher mean PCC than unidirectional and sum-pooling variants, and that shallow stacks (L=1 or L=2) with moderate $d_{\mathrm{model}}$ were sufficient. On that panel, residual-off L=1 had a higher mean PCC (0.6387) than residual-on L=1 (0.6191); Table 3 reports the residual-on L=1 reference configuration, and SoyBase oil and R8 in Table 5 used L=2. Residual-off was not substituted into the main comparison tables.

Computationally, BiMSGP is expensive to train relative to DeepGS (42 M versus 81 K parameters on Wheat599; 174 and 462 min per 100 epochs on Wheat2000 and SoyBase). That training cost is a genuine limitation for routine refitting. Millisecond inference is the benefit after a model has been trained.

Several additional limitations should be noted. A limitation of the evaluation is that the held-out folds were reused for checkpoint selection, learning-rate adjustment (ReduceLROnPlateau), and performance assessment. This may produce optimistic performance estimates and affect the apparent differences between models. The reported comparisons should therefore be interpreted as exploratory. Further evaluation separating model selection from final assessment is needed to establish generalization performance and confirm the comparative findings. Second, marker order followed source-file column order; chromosome junctions in the serialized sequence are not biological adjacencies. Third, the study does not include multi-omics inputs or independent-population validation. Future work should include nested evaluation, independent breeding populations or environments, model compression, and multi-trait or genotype-by-environment extensions [14,33].

## 5. Conclusions

BiMSGP is a bidirectional Mamba-based model for genomic prediction of complex plant traits. Bidirectional scanning lets each marker use sequence context from both directions while retaining linear complexity; this is a modeling device for long-range marker dependencies, not a demonstration of biological epistasis. Under the reported 10-fold protocol, in which the held-out fold was also used for checkpoint selection and learning-rate adjustment, BiMSGP obtained the numerically highest mean PCC for many quantitative traits and competitive mean scores for SoyBase categorical traits. Several close comparisons with DeepGS or GBLUP did not have exploratory $p_{t}<0.05$, and GBLUP had a higher mean PCC for SoyBase oil. These scores are exploratory and may be optimistic; they are not unbiased estimates of generalization and do not establish superiority over existing methods. Shallow bidirectional configurations with moderate model dimensions had higher mean PCC than deeper or unidirectional alternatives on Wheat599 Environment 1 under the same protocol.

Training BiMSGP is substantially more expensive than lightweight baselines; millisecond inference is the operational benefit after training. Evaluation that separates model selection from assessment, independent-population or multi-environment tests, and model compression are important next steps.

## 6. Patents

The authors have filed a patent application related to the BiMSGP model proposed in this manuscript, which has been accepted for examination. The filing does not prevent academic use or public release of the research code and experiment settings.

## Figures and Tables

**Table 1:** Summary of the datasets used in this study. SoyBase *n* is the trait-specific complete-case count among 15,878 genotyped accessions in the processed 42,195-SNP matrix, not the 13,784 × 32,032 panel of Gao et al. Categorical class counts are in Table 2.

ID	Name	Sample Size	Markers	Traits	Marker Type
1	Wheat599	599	1279	GY (Env1, Env2, Env3, Env4)	DArT
2	Wheat2000	2000	33,709	GL, GW, GH, TKW, TW, SDS, GP, PHT	DArT
3	SoyBase	14,485–15,878	42,195	Categorical: FC, P_DENS, POD, ST	SNP
				Quantitative: Hgt, Oil, Protein, SdWgt, R1, R8, Yield	

**Table 2:** Class counts for SoyBase categorical traits after excluding accessions with missing labels. Proportions are within each trait.

Trait	Classes	*n*	Class Counts (Proportion)
FC	W / P (binary)	15,731	W: 4844 (30.8%) P: 10,887 (69.2%)
P_DENS	Ssp / N (binary)	15,697	Ssp: 5720 (36.4%) N: 9977 (63.6%)
POD	Tn / Br / Bl (3-class)	15,490	Tn: 3863 (24.9%) Br: 9821 (63.4%) Bl: 1806 (11.7%)
ST	N / S / D (3-class)	15,164	N: 5907 (39.0%) S: 1225 (8.1%) D: 8032 (53.0%)

**Table 3:** Predictive performance on the Wheat599 dataset across four environments. Values are mean PCC ± standard deviation (SD), with RMSE ± SD shown below, over 10-fold cross-validation. For each trait and metric, boldface indicates the numerically best mean across models and does not indicate statistical significance (Table A1). Full BiMSGP settings, including the wide decoder and batch size 8, are in Section 2 (Implementation Details). PCC values are shown in top, while RMSE values are shown in bottom in each cell.

Method	Env 1	Env 2	Env 3	Env 4
GBLUP	0.5234 ± 0.0798 0.8521 ± 0.0419	0.5181 ± 0.0746 **0.8533 ± 0.0902**	0.4187 ± 0.0906 **0.9076 ± 0.1335**	0.4604 ± 0.0911 0.8855 ± 0.0960
DeepGS	0.5720 ± 0.0763 0.8220 ± 0.0668	0.5087 ± 0.1145 0.8757 ± 0.0766	0.4262 ± 0.0977 0.9306 ± 0.1393	0.5153 ± 0.1137 0.8765 ± 0.1242
DNNGP	0.5543 ± 0.1015 0.8397 ± 0.0641	0.5185 ± 0.0897 0.8868 ± 0.0829	0.4569 ± 0.0750 0.9265 ± 0.1127	0.5019 ± 0.1112 0.9144 ± 0.1060
SoyDNGP	0.5225 ± 0.1209 0.9927 ± 0.2207	0.4505 ± 0.1036 0.9876 ± 0.1200	0.4340 ± 0.1143 1.0875 ± 0.4172	0.5242 ± 0.0704 0.9315 ± 0.1146
BiMSGP	**0.6198 ± 0.0708** **0.8038 ± 0.0591**	**0.5626 ± 0.0834** 0.8693 ± 0.0872	**0.4836 ± 0.0701** 0.9456 ± 0.1458	**0.5732 ± 0.1079** **0.8485 ± 0.0974**

**Table 4:** Predictive performance on the Wheat2000 dataset for eight agronomic traits. Values are mean PCC ± SD, with RMSE ± SD shown below, over 10-fold cross-validation. For each trait and metric, boldface indicates the numerically best mean across models and does not indicate statistical significance (Table A2). Full BiMSGP settings, including the wide decoder and batch size 8, are in Section 2 (Implementation Details). PCC values are shown in top, while RMSE values are shown in bottom in each cell.

**Method**	**GH**	**GL**	**GP**	**GW**
GBLUP	0.6843 ± 0.0398 0.7347 ± 0.0378	0.7290 ± 0.0451 0.6888 ± 0.0470	0.5520 ± 0.0364 **0.8333 ± 0.0352**	0.7382 ± 0.0377 0.6778 ± 0.0647
DeepGS	0.6975 ± 0.0416 0.7224 ± 0.0421	0.7443 ± 0.0424 0.6728 ± 0.0487	0.5490 ± 0.0368 0.8642 ± 0.0905	**0.7500 ± 0.0331** 0.6679 ± 0.0592
DNNGP	0.4249 ± 0.0658 0.9876 ± 0.0299	0.4627 ± 0.0480 0.9902 ± 0.0770	0.4249 ± 0.0490 0.9952 ± 0.0358	0.5368 ± 0.0635 0.9756 ± 0.0497
SoyDNGP	0.5226 ± 0.0709 0.8742 ± 0.0456	0.6440 ± 0.0494 0.7738 ± 0.0465	0.4506 ± 0.0349 0.9179 ± 0.0576	0.6126 ± 0.0668 0.8272 ± 0.0736
BiMSGP	**0.6991 ± 0.0420** **0.7145 ± 0.0389**	**0.7530 ± 0.0434** **0.6563 ± 0.0433**	**0.5545 ± 0.0340** 0.8481 ± 0.0547	0.7464 ± 0.0384 **0.6655 ± 0.0668**
**Method**	**PHT**	**SDS**	**TKW**	**TW**
GBLUP	0.3236 ± 0.0769 **0.9440 ± 0.0527**	0.5333 ± 0.0411 **0.8459 ± 0.0334**	0.6609 ± 0.0296 0.7544 ± 0.0409	0.6194 ± 0.0611 0.7855 ± 0.0436
DeepGS	**0.3279 ± 0.0617** 0.9474 ± 0.0473	0.5285 ± 0.0435 0.8536 ± 0.0347	0.6751 ± 0.0313 **0.7400 ± 0.0393**	0.6302 ± 0.0632 0.7805 ± 0.0457
DNNGP	0.2311 ± 0.0459 0.9974 ± 0.0627	0.3504 ± 0.0599 0.9956 ± 0.0428	0.4252 ± 0.0554 0.9911 ± 0.0358	0.4378 ± 0.0468 0.9806 ± 0.0543
SoyDNGP	0.2248 ± 0.0527 0.9901 ± 0.0723	0.3575 ± 0.0586 0.9937 ± 0.0987	0.5792 ± 0.0590 0.8314 ± 0.0545	0.4970 ± 0.0906 0.9344 ± 0.1261
BiMSGP	0.3212 ± 0.0674 0.9592 ± 0.0502	**0.5401 ± 0.0353** 0.8504 ± 0.0368	**0.6760 ± 0.0327** 0.7476 ± 0.0423	**0.6343 ± 0.0520** **0.7763 ± 0.0389**

**Table 5:** Predictive performance on soybean quantitative traits. Values are mean PCC ± SD, with RMSE ± SD shown below, over 10-fold cross-validation. For each trait and metric, boldface indicates the numerically best mean across models and does not indicate statistical significance (Table A3). Full BiMSGP settings, including the compact decoder, batch size 32, and oil/R8 L=2 for 20 epochs, are in Section 2 (Implementation Details). PCC values are shown in top, while RMSE values are shown in bottom in each cell.

**Method**	**Hgt**	**Oil**	**Protein**	**SdWgt**
GBLUP	0.8077 ± 0.0131 **17.3797 ± 0.4693**	**0.8825 ± 0.0076** **1.2277 ± 0.0218**	**0.7182 ± 0.0105** **1.9667 ± 0.0633**	0.9167 ± 0.0068 2.1764 ± 0.0764
DeepGS	0.8073 ± 0.0152 17.6109 ± 0.7131	0.8804 ± 0.0082 1.2546 ± 0.0337	0.7101 ± 0.0100 2.0057 ± 0.0529	0.9187 ± 0.0069 2.2147 ± 0.0522
DNNGP	0.7884 ± 0.0189 18.2717 ± 0.5506	0.8738 ± 0.0073 1.2883 ± 0.0287	0.6961 ± 0.0122 2.0486 ± 0.0605	0.9117 ± 0.0075 2.2663 ± 0.0709
SoyDNGP	0.7385 ± 0.0187 31.8739 ± 6.3753	0.8301 ± 0.0117 7.4110 ± 1.4514	0.6450 ± 0.0122 13.2460 ± 2.4886	0.8768 ± 0.0071 11.4385 ± 2.1095
BiMSGP	**0.8141 ± 0.0135** 17.3879 ± 0.6601	0.8816 ± 0.0078 1.2456 ± 0.0236	0.7171 ± 0.0107 2.0118 ± 0.0731	**0.9201 ± 0.0073** **2.1498 ± 0.0871**
**Method**	**R1**	**R8**	**Yield**	
GBLUP	0.6753 ± 0.0095 45.1964 ± 0.5061	0.7755 ± 0.0184 38.2370 ± 1.2049	0.7861 ± 0.0127 **0.4582 ± 0.0108**	
DeepGS	0.6635 ± 0.0254 46.3535 ± 1.4084	0.7761 ± 0.0154 38.5956 ± 0.9967	0.7875 ± 0.0120 0.4621 ± 0.0149	
DNNGP	0.6648 ± 0.0129 46.1224 ± 0.8387	0.7686 ± 0.0151 38.9624 ± 0.8739	0.7744 ± 0.0084 0.4738 ± 0.0075	
SoyDNGP	0.5886 ± 0.0180 **41.6439 ± 11.2156**	0.6348 ± 0.2311 38.7465 ± 16.2311	0.7159 ± 0.0197 0.6253 ± 0.04853	
BiMSGP	**0.6821 ± 0.0114** 45.5045 ± 0.9148	**0.7846 ± 0.0176** **37.9279 ± 1.1924**	**0.7900 ± 0.0093** 0.4600 ± 0.0090	

**Table 6:** Classification performance on soybean categorical traits. Values are mean accuracy ± SD, with balanced accuracy ± SD shown below, over 10-fold StratifiedKFold cross-validation. For each trait and metric, boldface indicates the numerically best mean across models and does not indicate statistical significance. These metrics were not included in the paired PCC tests. Full BiMSGP settings, including the compact decoder and batch size 1, are in Section 2 (Implementation Details). Accuracy values are shown in top, while balanced accuracy values are shown in bottom in each cell.

Method	FC (Binary)	P_DENS (Binary)	POD (3-Class)	ST (3-Class)
GBLUP	0.9749 ± 0.0046 0.9696 ± 0.0062	0.8815 ± 0.0090 0.8684 ± 0.0095	0.8473 ± 0.0079 0.7749 ± 0.0111	0.7090 ± 0.0122 0.6595 ± 0.0220
DeepGS	0.9718 ± 0.0032 0.9663 ± 0.0055	0.8732 ± 0.0088 0.8623 ± 0.0128	0.8714 ± 0.0088 0.8462 ± 0.0141	**0.8203 ± 0.0093** **0.6697 ± 0.0222**
DNNGP	0.7198 ± 0.0832 0.5471 ± 0.1413	0.6575 ± 0.0658 0.5363 ± 0.1089	0.6575 ± 0.0707 0.3822 ± 0.1465	0.5624 ± 0.0930 0.3650 ± 0.0905
SoyDNGP	**0.9775 ± 0.0033** **0.9739 ± 0.0038**	**0.8838 ± 0.0072** **0.8731 ± 0.0103**	**0.8904 ± 0.0104** 0.8415 ± 0.0162	0.7973 ± 0.0131 0.6457 ± 0.0201
BiMSGP	0.9704 ± 0.0027 0.9650 ± 0.0043	0.8755 ± 0.0062 0.8649 ± 0.0077	0.8717 ± 0.0063 **0.8497 ± 0.0123**	0.8179 ± 0.0073 0.6695 ± 0.0220

**Table 7:** Computational cost of different BiMSGP configurations on the Wheat599 dataset. Training time is reported for 100 epochs.

Configuration	Parameter Count	Training Time (100 Epochs)	Inference Time (per Sample)
Lightweight Forward Mamba (L=1, d=64)	21.0 M	0.96 min	1.89 ms
Mamba (L=1, d=64)	42.0 M	1.22 min	1.64 ms
Mamba (L=2, d=64)	42.1 M	1.64 min	1.81 ms
Mamba (L=2, d=128)	168.4 M	3.17 min	1.86 ms
Mamba (L=2, d=256)	673.1 M	8.28 min	1.98 ms

**Table 8:** Ablation results for residual connections and number of layers ($d_{\mathrm{model}}=64$, $d_{\mathrm{state}}=64$, batch size 16). Values are mean PCC, RMSE, and MAE ± SD over 10-fold cross-validation. The best value for each metric is shown in bold. The residual-on L=1 main-table result uses batch size 8 and is reported separately in Table 3.

Variant	Layers	Residual	Avg. PCC	Avg. RMSE	Avg. MAE
Mamba (no res)	1	No	**0.6387 ± 0.0736**	0.7783 ± 0.0557	0.5978 ± 0.0516
Mamba (no res)	2	No	0.6378 ± 0.0676	0.7765 ± 0.0477	**0.5964 ± 0.0461**
Mamba (no res)	4	No	0.6374 ± 0.0651	**0.7738 ± 0.0620**	0.6041 ± 0.0479
Mamba (no res)	10	No	0.6321 ± 0.0734	0.7938 ± 0.0444	0.6144 ± 0.0458
Mamba	1	Yes	0.6191 ± 0.0708	0.7921 ± 0.0479	0.6076 ± 0.0398
Mamba	2	Yes	0.6295 ± 0.0772	0.7865 ± 0.0607	0.6027 ± 0.0492
Mamba	4	Yes	0.6271 ± 0.0834	0.7995 ± 0.0662	0.6131 ± 0.0467
Mamba	10	Yes	0.6259 ± 0.0707	0.7962 ± 0.0647	0.6133 ± 0.0562

**Table 9:** Ablation results for model dimension, state dimension, and architectural variants (fixed L=2). Values include trainable parameters and mean PCC, RMSE, and MAE ± SD over 10-fold cross-validation. The best value for each metric is shown in bold.

Variant	$d_{\mathrm{model}}$	$d_{\mathrm{state}}$	Parameter Count	Avg. PCC	Avg. RMSE	Avg. MAE
Mamba	32	64	10.6 M	0.6197 ± 0.0774	0.7960 ± 0.0560	0.6131 ± 0.0441
Mamba	64	64	42.1 M	0.6295 ± 0.0772	0.7865 ± 0.0607	0.6027 ± 0.0492
Mamba	128	64	168.4 M	**0.6387 ± 0.0835**	0.7867 ± 0.0760	0.6005 ± 0.0543
Mamba	256	64	673.1 M	0.6364 ± 0.0663	0.7906 ± 0.0754	0.6040 ± 0.0493
Mamba	64	16	42.1 M	0.6242 ± 0.0829	0.7919 ± 0.0616	0.6029 ± 0.0506
Mamba	64	32	42.1 M	0.6337 ± 0.0665	**0.7803 ± 0.0479**	**0.5932 ± 0.0314**
ForwardMamba	64	64	21.1 M	0.6215 ± 0.0742	0.8052 ± 0.0652	0.6121 ± 0.0420
BackwardMamba	64	64	21.1 M	0.6224 ± 0.0767	0.7997 ± 0.0528	0.6064 ± 0.0400
Mamba (sum)	64	64	21.2 M	0.6232 ± 0.0834	0.7985 ± 0.0737	0.6092 ± 0.0596

**Table 10:** Computational efficiency comparison on the Wheat599 dataset. Training time is reported for 100 epochs.

Model	Parameter Count	Training Time (100 Epochs)	Inference Time (per Sample)
BiMSGP	42 M	1.52 min	1.7 ms
DeepGS	81 K	0.40 min	1.1 ms
DNNGP	10.4 M	0.48 min	1.3 ms
SoyDNGP	18.1 M	0.80 min	1.4 ms
GBLUP	–	<0.1 s/fold	–

## Data Availability

The datasets analyzed in this study are publicly available from the original publications and repositories cited in the Materials and Methods section. The research implementation is publicly available at https://github.com/liuxumail-create/BiMSGP (accessed on 17 September 2026) and provides the trainer, JSON configs, fold-export helpers, published fold assignments, and fold-wise metrics. Regression used KFold and SoyBase classification used StratifiedKFold (n_splits = 10, shuffle = True, random_state = 42). The public code reads already-processed genotype matrices; scripts that rebuild the 1279-marker Wheat599 table or the 42,195-column SoyBase table from raw downloads are not included. The patent application related to BiMSGP does not restrict academic release of the implementation.

## Abbreviations

**Abbreviation**	**Definition**
BiMSGP	Bidirectional Mamba-based Genomic Prediction
Mamba	Selective State Space Model
SSM	State Space Model
SNP	Single Nucleotide Polymorphism
DArT	Diversity Arrays Technology
PCC	Pearson Correlation Coefficient
RMSE	Root Mean Squared Error
MAE	Mean Absolute Error
GBLUP	Genomic Best Linear Unbiased Prediction
DeepGS	Deep Genomic Selection
DNNGP	Deep Neural Network Genomic Prediction
SoyDNGP	Soybean Deep Neural Network Genomic Prediction
CV	Cross-Validation

## Appendix A. Paired Fold-Wise Tests

Table A1, Table A2 and Table A3 report exploratory paired comparisons of fold-wise PCC (BiMSGP minus the comparator) on the ten shared cross-validation folds. The 95% CI is a Student’s *t* interval for the mean fold-wise difference. $p_{t}$ is the primary exploratory test; $p_{W}$ is supplementary. *p*-values are unadjusted, and overlapping training folds mean that the ten differences are not independent. RMSE and classification metrics are not included. SoyDNGP fold-level scores were unavailable for Wheat599 and for SoyBase quantitative traits.

**Table A1:** Exploratory paired fold-wise PCC comparisons of BiMSGP versus competing methods on Wheat599 (10 shared folds). ΔPCC is the mean of fold-wise differences (BiMSGP minus the comparator). The 95% CI is a Student’s *t* interval for that mean (df =9). $p_{t}$ is a two-sided paired *t*-test (primary exploratory test); $p_{W}$ is a two-sided Wilcoxon signed-rank test (supplementary). $d_{z}=\bar{\Delta }/s_{\Delta }$. Stars refer to unadjusted $p_{t}$: * p<0.05, ** p<0.01, *** p<0.001; ns, $p_{t}\geq 0.05$. Overlapping CV training folds violate independence; *p*-values are unadjusted and Holm correction was not applied. SoyDNGP fold-level scores were not archived for this dataset.

Trait	Comparator	ΔPCC	95% CI	$p_{t}$	$p_{W}$	$d_{z}$	Sig.
Env1	DeepGS	+0.0478	[+0.0029, +0.0926]	0.039	0.037	0.76	*
	DNNGP	+0.0655	[+0.0048, +0.1261]	0.037	0.049	0.77	*
	GBLUP	+0.0964	[+0.0640, +0.1288]	<0.001	0.002	2.13	***
Env2	DeepGS	+0.0538	[−0.0087, +0.1164]	0.083	0.131	0.62	ns
	DNNGP	+0.0441	[+0.0076, +0.0806]	0.023	0.027	0.86	*
	GBLUP	+0.0445	[+0.0177, +0.0713]	0.004	0.004	1.19	**
Env3	DeepGS	+0.0574	[+0.0080, +0.1068]	0.027	0.027	0.83	*
	DNNGP	+0.0268	[−0.0107, +0.0643]	0.141	0.193	0.51	ns
	GBLUP	+0.0649	[+0.0243, +0.1056]	0.006	0.010	1.14	**
Env4	DeepGS	+0.0579	[−0.0092, +0.1250]	0.083	0.131	0.62	ns
	DNNGP	+0.0713	[+0.0298, +0.1128]	0.004	0.004	1.23	**
	GBLUP	+0.1128	[+0.0614, +0.1642]	<0.001	0.002	1.57	***

**Table A2:** Exploratory paired fold-wise PCC comparisons of BiMSGP versus competing methods on Wheat2000 (all eight traits; 10 shared folds). Notation as in Table A1.

Trait	Comparator	ΔPCC	95% CI	$p_{t}$	$p_{W}$	$d_{z}$	Sig.
GH	DeepGS	+0.0016	[−0.0056, +0.0088]	0.629	0.770	0.16	ns
	DNNGP	+0.2743	[+0.2348, +0.3137]	<0.001	0.002	4.98	***
	GBLUP	+0.0149	[+0.0094, +0.0203]	<0.001	0.002	1.96	***
	SoyDNGP	+0.1766	[+0.1370, +0.2161]	<0.001	0.002	3.19	***
GL	DeepGS	+0.0087	[−0.0018, +0.0192]	0.093	0.105	0.59	ns
	DNNGP	+0.2903	[+0.2553, +0.3253]	<0.001	0.002	5.93	***
	GBLUP	+0.0240	[+0.0188, +0.0292]	<0.001	0.002	3.30	***
	SoyDNGP	+0.1090	[+0.0874, +0.1306]	<0.001	0.002	3.61	***
GP	DeepGS	+0.0055	[−0.0082, +0.0192]	0.385	0.432	0.29	ns
	DNNGP	+0.1295	[+0.0880, +0.1710]	<0.001	0.002	2.23	***
	GBLUP	+0.0024	[−0.0056, +0.0105]	0.511	0.625	0.22	ns
	SoyDNGP	+0.1039	[+0.0904, +0.1174]	<0.001	0.002	5.52	***
GW	DeepGS	−0.0036	[−0.0115, +0.0042]	0.325	0.432	−0.33	ns
	DNNGP	+0.2096	[+0.1834, +0.2358]	<0.001	0.002	5.72	***
	GBLUP	+0.0082	[+0.0026, +0.0138]	0.009	0.014	1.05	**
	SoyDNGP	+0.1337	[+0.0860, +0.1815]	<0.001	0.002	2.00	***
PHT	DeepGS	−0.0068	[−0.0272, +0.0137]	0.473	0.492	−0.24	ns
	DNNGP	+0.0901	[+0.0596, +0.1205]	<0.001	0.002	2.12	***
	GBLUP	−0.0024	[−0.0199, +0.0152]	0.767	0.922	−0.10	ns
	SoyDNGP	+0.0964	[+0.0481, +0.1447]	0.001	0.006	1.43	**
SDS	DeepGS	+0.0116	[−0.0064, +0.0296]	0.179	0.232	0.46	ns
	DNNGP	+0.1897	[+0.1516, +0.2278]	<0.001	0.002	3.56	***
	GBLUP	+0.0068	[−0.0023, +0.0160]	0.126	0.131	0.53	ns
	SoyDNGP	+0.1826	[+0.1353, +0.2300]	<0.001	0.002	2.76	***
TKW	DeepGS	+0.0008	[−0.0081, +0.0098]	0.837	0.922	0.07	ns
	DNNGP	+0.2507	[+0.2212, +0.2803]	<0.001	0.002	6.08	***
	GBLUP	+0.0151	[+0.0060, +0.0242]	0.005	0.004	1.18	**
	SoyDNGP	+0.0968	[+0.0650, +0.1286]	<0.001	0.002	2.18	***
TW	DeepGS	+0.0042	[−0.0121, +0.0205]	0.575	0.695	0.18	ns
	DNNGP	+0.1966	[+0.1667, +0.2265]	<0.001	0.002	4.71	***
	GBLUP	+0.0149	[+0.0032, +0.0266]	0.018	0.020	0.91	*
	SoyDNGP	+0.1373	[+0.0796, +0.1951]	<0.001	0.002	1.70	***

**Table A3:** Exploratory paired fold-wise PCC comparisons of BiMSGP versus competing methods on SoyBase quantitative traits (10 shared folds). Notation as in Table A1. SoyDNGP fold-level scores were not archived for these traits. Classification accuracy and balanced accuracy were not tested.

Trait	Comparator	ΔPCC	95% CI	$p_{t}$	$p_{W}$	$d_{z}$	Sig.
Hgt	DeepGS	+0.0068	[+0.0019, +0.0116]	0.011	0.014	1.00	*
	DNNGP	+0.0257	[+0.0183, +0.0331]	<0.001	0.002	2.48	***
	GBLUP	+0.0064	[+0.0036, +0.0091]	<0.001	0.004	1.64	***
Oil	DeepGS	+0.0011	[−0.0000, +0.0023]	0.058	0.064	0.68	ns
	DNNGP	+0.0077	[+0.0058, +0.0096]	<0.001	0.002	2.90	***
	GBLUP	−0.0010	[−0.0018, −0.0002]	0.025	0.020	−0.85	*
Protein	DeepGS	+0.0070	[−0.0002, +0.0143]	0.056	0.084	0.69	ns
	DNNGP	+0.0210	[+0.0127, +0.0293]	<0.001	0.002	1.82	***
	GBLUP	−0.0011	[−0.0078, +0.0055]	0.706	0.492	−0.12	ns
SdWgt	DeepGS	+0.0014	[−0.0002, +0.0030]	0.076	0.105	0.63	ns
	DNNGP	+0.0084	[+0.0058, +0.0110]	<0.001	0.002	2.31	***
	GBLUP	+0.0033	[+0.0022, +0.0045]	<0.001	0.004	2.14	***
R1	DeepGS	+0.0186	[+0.0028, +0.0345]	0.026	0.002	0.84	*
	DNNGP	+0.0173	[+0.0119, +0.0227]	<0.001	0.002	2.29	***
	GBLUP	+0.0067	[+0.0001, +0.0134]	0.047	0.131	0.73	*
R8	DeepGS	+0.0085	[+0.0031, +0.0139]	0.006	0.002	1.13	**
	DNNGP	+0.0160	[+0.0114, +0.0207]	<0.001	0.002	2.46	***
	GBLUP	+0.0091	[+0.0059, +0.0124]	<0.001	0.002	1.99	***
Yield	DeepGS	+0.0025	[−0.0015, +0.0065]	0.193	0.193	0.45	ns
	DNNGP	+0.0156	[+0.0136, +0.0176]	<0.001	0.002	5.65	***
	GBLUP	+0.0039	[−0.0005, +0.0082]	0.074	0.105	0.64	ns

## Appendix B. Computational Efficiency on Larger Datasets

Training times on Wheat2000 and SoyBase were substantially longer than on Wheat599, primarily because of BiMSGP’s parameter count (1.105 billion on Wheat2000 and 691 million on SoyBase, compared with 42 million on Wheat599 and 81K for DeepGS on Wheat599). This capacity may help represent long marker sequences, but it is a real training-cost disadvantage.

Detailed results are presented in Table A4. On Wheat2000, BiMSGP required approximately 174 min for 100 epochs, and on SoyBase approximately 462 min, versus under 10 min for DeepGS on those panels. Inference remained in the millisecond range, which is relevant after a model has already been trained rather than as a substitute for the training cost.

In future work, we plan to explore shallower network architectures to reduce the parameter count and training time while aiming to preserve the predictive advantages of the current BiMSGP model.

**Table A4:** Computational efficiency comparison of BiMSGP and competing methods across three datasets.

Dataset	Model	Parameter Count	Training Time (100 Epochs)	Inference Time (per Sample)
Wheat599 (n=599, m=1279)	BiMSGP	42 M	1.52 min	1.7 ms
	DeepGS	81 K	0.40 min	1.1 ms
	DNNGP	10.4 M	0.48 min	1.3 ms
	SoyDNGP	18.1 M	0.80 min	1.4 ms
	GBLUP	–	<0.1 s/fold	–
Wheat2000 (n=2000, m=33,709)	BiMSGP	1.105 B	174 min	11.9 ms
	DeepGS	8.6 M	0.98 min	0.63 ms
	DNNGP	276 M	5.3 min	0.87 ms
	SoyDNGP	18.1 M	2.8 min	0.73 ms
	GBLUP	–	0.2 s/fold	–
SoyBase (trait-specific n=14,485–15,878; 42,195 SNPs)	BiMSGP	691 M	462 min	4.9 ms
	DeepGS	21.6 M	9 min	0.5 ms
	DNNGP	346 M	37–43 min	0.6 ms
	SoyDNGP	18.4 M	80 min	3 ms
	GBLUP	–	6–7 s/fold	≈4 ms

## Appendix C. Bar-Plot Summaries of Main-Table Results

Figure A1, Figure A2, Figure A3 and Figure A4 are bar-plot summaries of the primary metric in Table 3, Table 4, Table 5 and Table 6. They were moved out of the Results section so that the main text does not repeat the same comparisons in both a table and a figure. Exact mean ± SD values remain in the main tables, and the paired tests remain in Appendix A.
